# Bio-Based Hydrogels With Ion Exchange Properties Applied to Remove Cu(II), Cr(VI), and As(V) Ions From Water

**DOI:** 10.3389/fbioe.2021.656472

**Published:** 2021-05-20

**Authors:** Julio Sánchez, Daniel Dax, Yesid Tapiero, Chunlin Xu, Stefan Willför

**Affiliations:** ^1^Departamento de Ciencias del Ambiente, Facultad de Química y Biología, Universidad de Santiago de Chile, Santiago, Chile; ^2^Research Group of Wood and Paper Chemistry, Laboratory of Natural Materials Technology, Åbo Akademi University, Turku, Finland

**Keywords:** arsenic, hemicellulose, hydrogel, metal ions, water treatment

## Abstract

Hydrogels with ion exchange properties were synthesized from compounds derived from wood biopolymer hemicellulose and from commercial vinyl monomers to be tested as active materials for the removal of Cu(II), Cr(VI), and As(V) ions. The hemicellulose *O*-acetyl galactoglucomannan (GGM) was used as the precursor material, and through a transesterification reaction, GGM was converted into a macromonomer GGM–glycidyl methacrylate (GGM-GMA). Subsequently, the GGM-GMA macromonomer, containing more than one methacrylate group, was used as a crosslinking agent in the synthesis of hydrogels through free-radical polymerization reactions in combination with a 2-acrylamido-2-methyl-1-propanesulfonic acid monomer to produce a cation exchange hydrogel. Also, (3-acrylamidopropyl)trimethylammonium chloride monomer was applied together with the GGM-GMA to form hydrogels that can be used as anion exchange hydrogel. The hydrogels were characterized by Fourier transform-infrared (FT-IR), ^1^H-NMR spectroscopy, and thermogravimetric analysis (TGA), as well as derivative thermogravimetry (DTG). The microstructure of the hydrogels was characterized by scanning electron microscopy (SEM) analysis with X-ray microanalysis energy-dispersive spectroscopy (EDS). The results obtained regarding the absorption capacity of the Cu(II), Cr(VI), and As(V) ions were studied as a function of the pH value and the initial concentration of the metal ions in the solutions. Absorption was carried out in consecutive batches, and it was found that the poly(GGM-GMA/AMPSH) hydrogel reached an absorption capacity of 90 mg g^–1^ for Cu(II). The poly(GGM-GMA/APTACl) hydrogel reached values of 69 and 60 mg g^–1^ for Cr(VI) and As(V) oxyanions, respectively. Tests with polymer blends (mixtures of anionic and cationic hydrogels) were also carried out to remove Cu(II), Cr(VI), and As(V) ions from multi-ionic solutions, obtaining satisfactory results.

## Introduction

An important material in industry is wood, as it plays a key role in the development of anthropogenic activities at all levels. The main applications of wood include transportation systems, energy, industrial and residential buildings, and works of art, among others. However, as a biomaterial, wood often undergoes decomposition by biotic, chemical, or physical agents. For this reason, different strategies have been designed to extend the useful life of wood through chemical or physical treatments. Water-based chemical treatments are the most commonly used due to their simplicity and because they are economical; of this type, the preservative formulation based on a copper–chrome–arsenic (CCA) mixture stands out (Ohgami et al., 2015). This mixture is generally applied to wood by a vacuum pressure impregnation method. CCA can be classified into three different types: type A (16.4% arsenic, 18.0% copper, and 65.5% chromium), type B (45.1% arsenic, 19.6% copper, and 35.3% chromium), or type C (34.0% arsenic, 18.5% copper, and 47.5% chromium) (Janin et al., 2009a; Coudert et al., 2014; Ohgami et al., 2015). The function of copper is bactericidal and fungicidal, arsenic is an insecticide, and chromium is the binding agent for wood, copper, and arsenic (Zheng et al., 2015; Gezer and Cooper, 2016).

Wood treated with the preservative (CCA) has been shown to last a long time; however, depending on the environmental conditions, release phenomena of arsenic, copper, and chromium can be generated from wood, which can cause serious health problems, as these elements have toxic effects on the cell cycle (Matos et al., 2020). The CCA wood preservative has permitted uses in commercial, industrial, and agricultural activities (Matos et al., 2020), where the components of the preservatives are easily leached, contaminating soil, water, and plants and causing harm to animals and humans. CCA contamination occurs through dermatological absorption, inhalation of wood dust, or contact with contaminated water and soil (Zheng et al., 2015; Safa et al., 2020).

Depending on where wood is used, certain levels of CCA application are required; for example, on land, 4.0 kg m^–3^ of CCA is needed, and for applications at sea, 40.0 kg m^–3^ of CCA is needed (Gezer and Cooper, 2016). A large amount of CCA remains impregnated in wood until the end of its useful life, where it remains a problem as waste (Helsen and Van den Bulck, 2005). When wood treated with the CCA preservative is disposed of in a municipal landfill, where it mixes with common garbage and undergoes decomposition processes, it releases toxic chromium and arsenic ions, contaminating the leachate from waste. There is no waste regulation regarding CCA-treated wood waste in the United States, Canada, or other countries (Janin et al., 2009b). The danger of leachates produced from preservative-treated woods is that exposure to arsenic can generate free radicals and oxidative stress in cells and cause death (Chen and Olsen, 2016). Exposure to chromium is toxic due to its oxidative character (Saha and Orvig, 2010), and prolonged exposure to copper can cause carcinogenic effects (Ohgami et al., 2015). For this reason, the World Health Organization recommends the following maximum concentrations in water: 0.01 mg L^–1^ for arsenic, 0.05 mg L^–1^ for chromium, and 2.0 mg L^–1^ for copper (Tyagi et al., 2013).

CCA leachates are conventionally treated with chemical methods (oxidizing agents, chelators, and organic and inorganic acids) (Almaroai et al., 2013), chelating polymers (chitin, chitosan, and biomaterials) (Dhillon et al., 2017; Wilson and Tewari, 2018), biodegradation and/or bioleaching with bacteria and fungi (Sierra-Alvarez, 2009), ion exchange processes with resins and coagulation–precipitation techniques (Yan et al., 2020), extraction with hot water and dilute sulfuric acid (Ferrarini et al., 2016), and thermochemical conversion methods (Kramb et al., 2016), among others. The drawbacks of these common treatments are the excessive consumption of reactive material, long reaction times, and generation of toxic sludge. Sahiner et al. designed cryogels and hydrogels based on poly[(3-acrylamidopropyl)trimethylammonium chloride] [poly(APTACl)], which has a high surface porosity capacity, allowing for rapid water absorption, and has a maximum adsorption capacity of approximately 120 mg g^–1^ of As(V) oxyanions. The poly((3-acrylamidopropyl) trimethylammonium chloride) hydrogel can be reused five times in the adsorption of As(V) from aqueous environments. This hydrogel can be rapidly regenerated and does not produce highly hazardous toxic waste in large quantities as occurs in other water treatment processes (Sahiner et al., 2015).

Hydrogels are attractive materials for water treatment because, in some cases, they can be synthesized from biomaterials, and they can also be designed to have adjustable and tuneable properties, including the pH value and ionic strength, for use in an aqueous working medium (Kong et al., 2018). The most important consideration is to identify a raw material that provides a large amount of biocompounds to develop starting materials that can polymerize and produce hydrogels. A promising source is hemicellulose extracted from wood, the second most abundant polysaccharide after cellulose. A hemicellulose hydrogel that is pH-sensitive and partially biodegradable can be prepared by grafting vinyl monomers into a polysaccharide structure, specifically in acetyl bonds. *O*-Acetyl galactoglucomannan (GGM) is a form of hemicellulose and the major type in softwoods and is employed in the production of anionic, cationic, or neutral polysaccharide-based hydrogels. Hydrogels based on the GGM modification can remove heavy metals from aqueous solutions via adsorption. These gels can be reused for up to eight cycles (Farrukh et al., 2018). Likewise, hydrogels derived from cellulose and hemicellulose materials have been reported, which have found applications in the retention of heavy metal ions; for example, Dax et al. (2014) modified GGM by grafting glycidyl methacrylate (GMA) to generate a vinyl macromonomer that can be crosslinked with the monomer [2-(methacryloyloxy)ethyl]trimethylammonium chloride to create a hydrogel with the capacity to remove arsenic and chromium oxyanions at concentrations between 55 and 68 mg g^–1^ at a pH value of 9.0. In another study, the macromonomer GGM-GMA was crosslinked with 2-acrylamido-2-methyl-1-propanesulfonic acid (AMPSH), acrylic acid, and acrylamide to obtain ion exchange hydrogels. These materials were tested for the retention of metallic ions dissolved in water, such as cadmium, copper, lead, nickel, and zinc; lead was the cation with the greatest strength of interaction and retention in the structure of the hydrogels as compared with the other metallic ions (Elgueta et al., 2016). In all reported cases of the use of GGM crosslinked hydrogels, the removal of metal ions was studied in mono-ion solutions (anionic or cationic), not in mixtures or multi-ion solutions (anionic and cationic).

The aim of this research was to take advantage of the properties of GGM to produce a macromonomer, GGM-GMA, with the ability to generate ion exchange hydrogels to remove Cu(II), Cr(VI), and As(V) ions at various pH levels and concentrations and to study the removal of these ions from CCA multi-ion solutions.

## Experimental

### Materials

2-acrylamido-2-methyl-1-propanesulfonic acid, AMPSH (99%, Aldrich, Chile), (3-acrylamidopropyl)trimethylammonium chloride, APTACl solution (75% in water, Aldrich, Chile), dimethylsulfoxide (DMSO) (Merck, Chile), acetone (Merck, Chile), GMA (97%, Aldrich, Chile), (NH_4_)_2_S_2_O_8_ (98%, Aldrich, Chile), 4-(dimethylamino)pyridine (DMAP) (98%, Aldrich, Chile), ethanol (Merck, Chile), sodium hydroxide NaOH (Merck, Chile), nitric acid HNO_3_ (65%, Merck, Chile), sodium bisulfite NaHSO_3_ (98%, Aldrich, Chile), copper(II) nitrate trihydrate Cu(NO_3_)_2_⋅3H_2_O (98,9%, Merck, Chile), potassium chromate K_2_CrO_4_ (99, 9%, Merck, Chile), and sodium arsenate dibasic heptahydrate Na_2_HAsO_4_⋅7H_2_O (98%, Merck, Chile).

### Methods

#### Synthesis of the (*O*-Acetyl Galactoglucomannan–Glycidyl Methacrylate) Macromonomer

Pressurized hot water-extracted GGM from Norway spruce (*Picea abies*) was provided by The Finnish Forest Research Institute Metla. The extraction conditions were studied and optimized in a previous study (Dax et al., 2014; Elgueta et al., 2016).

The transesterification reaction to obtain macromonomer was performed in a three-necked round-bottom glass flask to introduce the reactive material. The reaction was carried out by mixing *O*-acetyl-galactoglucomannan (GGM: 10.0 g, 58.1 mmol) with GMA (5.9 g, 41.5 mmol). DMAP (10 mol% GMA) was added as the catalyst, and the reaction medium was DMSO. It was necessary to remove dissolved oxygen from the reagent mixture; therefore, nitrogen gas was injected for 15 min. The transesterification reaction was carried out at a temperature of 50°C for 72 h with constant stirring. At the end of the reaction, acetone was added to the mixture to precipitate the product, followed by a separation process through vacuum filtration. When the precipitated solid was completely dry, it was dissolved in 50 ml of distilled water, and the separation was refined using a dialysis membrane tube (cutoff of 3.5 kDa) for 3 days, with water exchange every 24 h. Finally, the retentate obtained inside the dialysis tube was lyophilized for 7 days (Dax et al., 2014; Elgueta et al., 2016).

#### Synthesis of Hydrogels

Synthesis of the hydrogels was carried out through free-radical polymerization using water as the reaction solvent at 60°C for 12 h. Two classes of hydrogels were prepared, one cationic and one anionic. The cationic hydrogel was labeled poly(GGM-GMA/APTACl), and the anionic hydrogel was labeled poly(GGM-GMA/AMPSH). Synthesis of both hydrogels was performed using the same procedure. In a flask, 0.8 g of the macromonomer (GGM-GMA) and 7.20 g of a vinyl monomer (APTACl or AMPSH) were mixed with 32 ml of water, 0.252 g of the radical initiator (NH_4_)_2_S_2_O_8_, and 0.249 g of the NaHSO_3_ oxygen scavenger. After the solid hydrogel was obtained, it was washed with bidistilled water in a flask with constant agitation for 12 h at room temperature. Subsequently, the samples were filtered under vacuum, continuing the lyophilization of the hydrogel until the sample was completely dry.

#### Characterization

A Bruker Avance 400-MHz spectrometer was used for the ^1^H-NMR spectra measurements, with D_2_O as the solvent and tetramethylsilane as the internal standard. A Nicolet Magna 550 spectrophotometer was used for the Fourier transform-infrared (FT-IR) measurements of the polymers in the spectral range of 4,000–400 cm^–1^. Polymer samples were prepared in KBr. A Thermobalance TG209 Iris F1^®^ was used for the thermogravimetric analysis (TGA). The analysis was performed under a nitrogen gas atmosphere with a heating rate of 10°C min^–1^ over a temperature range between 30 and 550°C. Scanning electron microscopy (SEM) (20,000 kV JOEL microscopy; JSH 6380 LV model) and an Oxford instruments INCAx-sight instrument were used for SEM–energy-dispersive spectroscopy (EDS) measurements.

#### Water Uptake

Polymer samples of 0.1 g (*m*_*dry^0*_) were exposed to 100 ml of bidistilled water. The aim was to study the hydrogel–water balance for 24 h at room temperature. The hydrogel was then vacuum filtered, and the weight of the hydrated hydrogel (*m*_*wet ^f*_) was recorded. The percentage of water uptake is calculated according to the following:

$$\%W=\frac{m_{w\,e\,t^{f}}-m_{d\,r\,y^{0}}}{m_{d\,r\,y^{0}}}\times 100\%$$

#### Removal of Metal Ions

For each metal ion removal experiment, 0.02 g of polymer was placed in 5 ml of a metal ion solution and then stirred at 180 rpm for 1 h at 25°C. The polymer–metal ion solution mixture was then centrifuged at 4,000 rpm for 20 min to separate the solid and liquid phases. Finally, the liquid supernatant was analyzed by atomic absorption spectroscopy (AAS) (Unicam Solaar 5 M series) to determine the metal ion concentration. For the AAS measurements, a flame atomizer with a mixture of 40% acetylene and 60% oxygen was used. An electrode discharge lamp was used as the radiation source.

To study the retention behavior of Cr(VI) and As(V) oxyanions, a cationic GGM-GMA/APTACl hydrogel was used. Cu(II) retention was studied with the anionic GGM-GMA/AMPSH hydrogel. For the experiment to determine the removal of mono-ion solutions as a function of pH, 100 mg L^–1^ of Cu(NO_3_)_2_⋅3H_2_O, 100 mg L^–1^ of K_2_CrO_4_, and 100 mg L^–1^ of Na_2_HAsO_4_⋅7H_2_O were prepared in separate solutions to study the removal of Cu(II), Cr(VI), and As(V), respectively. The pH values analyzed were 3.0, 6.0, and 9.0 [for Cr(VI) and As(V)], which were adjusted with HNO_3_ or NaOH standards. For Cu(II), the pH values analyzed were 2.0, 3.0, and 4.0. To study the retention of metal ions as a function of the metal concentration (50, 100, 200, and 300 mg L^–1^), Cr(VI) and As(V) were analyzed at pH 9.0, while Cu(II) was analyzed at pH 3.0. In the absorption–desorption studies, solutions of 100 mg L^–1^ of each metal ion were prepared (feed) at the optimum working pH. First, 0.02 g of the polymer was added to 10.0 ml of an aqueous solution containing the ion of interest. The hydrogel–metal ion solution was kept under constant agitation for 1 h at 180 rpm and 25°C and then centrifuged for 20 min at 4,000 rpm. Subsequently, the supernatant was removed, diluted to 25.0 ml with bidistilled water, and measured by AAS. The hydrogel material was then washed with 1 mol L^–1^ of NaCl brine to pH 6.7, followed by water washes. Subsequently, the hydrogel added to the same metal solution of the feed, and the process was repeated in three absorption–desorption cycles.

#### Mixed Bed Column Tests

The mixture of poly(GGM-GMA/APTACl) (0.02 g) and poly(GGM-GMA/AMPSH) (0.02 g) hydrogels was used as ion exchange extracting agents. The hydrogel mixture was then added to 10 ml of each mono-ionic aqueous solution [100 mg L^–1^ of Cu(II), Cr(VI), and As(V) in separate solutions] and at the optimal pH values. Similarly, the mixture of the ion exchange hydrogels was added to 10 ml of a multi-ionic aqueous solution containing 100 mg L^–1^ of each metal ion. All the absorption samples were placed in a stirred, thermoregulated bath at 25°C for 1 h, and then, the solution was centrifuged for approximately 20 min at 4,000 rpm. The supernatant was carefully removed with a micropipette and diluted to 25 ml. This procedure was applied in all the experimental tests.

The absorption capacity (*S*) of the hydrogels was evaluated using the following equation:

$$S=\frac{\left(C_{0}^{m\,e\,t\,a\,l}-C_{t}^{m\,e\,t\,a\,l}\right)\times V}{m_{d\,r\,y}^{H}}$$

where $m_{d\,r\,y}^{H}$ is the mass of the dried hydrogel (mg); $C_{0}^{m\,e\,t\,a\,l}$ and $C_{t}^{m\,e\,t\,a\,l}$ (mg/L) are the concentrations of the metal ions in the solution before and after sorption by the hydrogels, respectively; and *V* is the volume (L) of the aqueous phase.

## Results and Discussion

### Characterization

From the results of the transesterification reaction, a yield of over 47.3% was obtained for the formation of the macromonomer (GGM-GMA) with 4.732 g GGM as the starting material.

**FIGURE 1:**
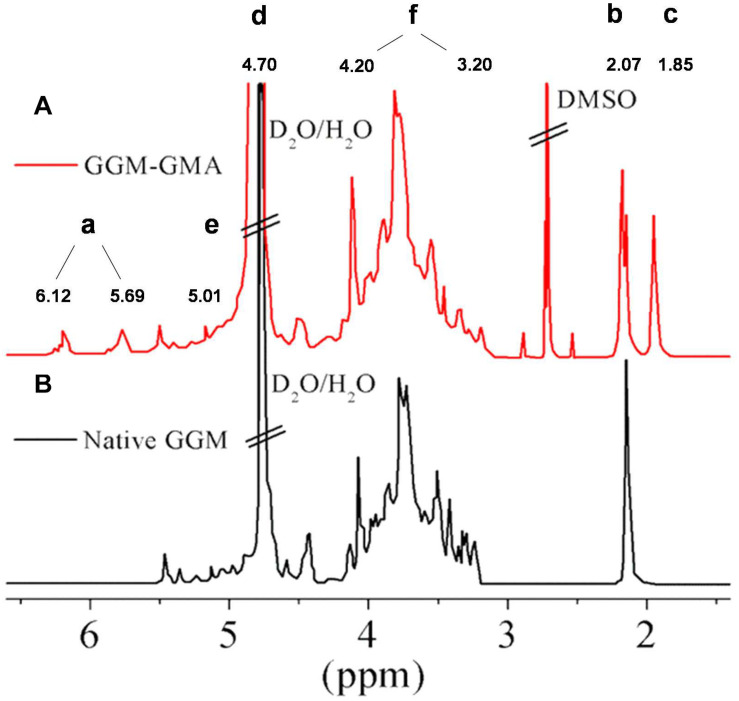
^1^H-NMR spectra of **(A)** GGM-GMA and **(B)** native GGM in D_2_O. GGM, *O*-acetyl galactoglucomannan; GMA, glycidyl methacrylate.

Figure 1 shows the signals of the spectrum obtained through ^1^H-NMR spectroscopy for the GGM-GMA macromonomer and native GGM. In the spectrum, signal (a) two peaks corresponding to the protons of the double bond –C=C– of the methacrylate group grafted through the transesterification reaction are observed. The peaks between 5.69 and 6.12 ppm correspond to native GGM, but they are small and poorly defined; however, it is observed that in GGM-GMA, they are separate and well-defined peaks. Signal (b) at 2.07 ppm corresponds to the protons of the solvent DMSO (Dax et al., 2014). Signal (c) at 1.85 ppm corresponds to two overlapping and sharp peaks of the methyl group. Signal (d) at 4.70 ppm is attributed to deuterated water, and signal (e) at 4.90 ppm corresponds to the anomeric protons of GGM-GMA. The protons of OC–OCH_3_ are observed at 2.86 and 2.64 ppm, and signal (f) between 3.20 and 4.20 ppm corresponds to the protons from H_1_ to H_6_ of GGM-GMA (Heinze et al., 2006).

**FIGURE 2:**
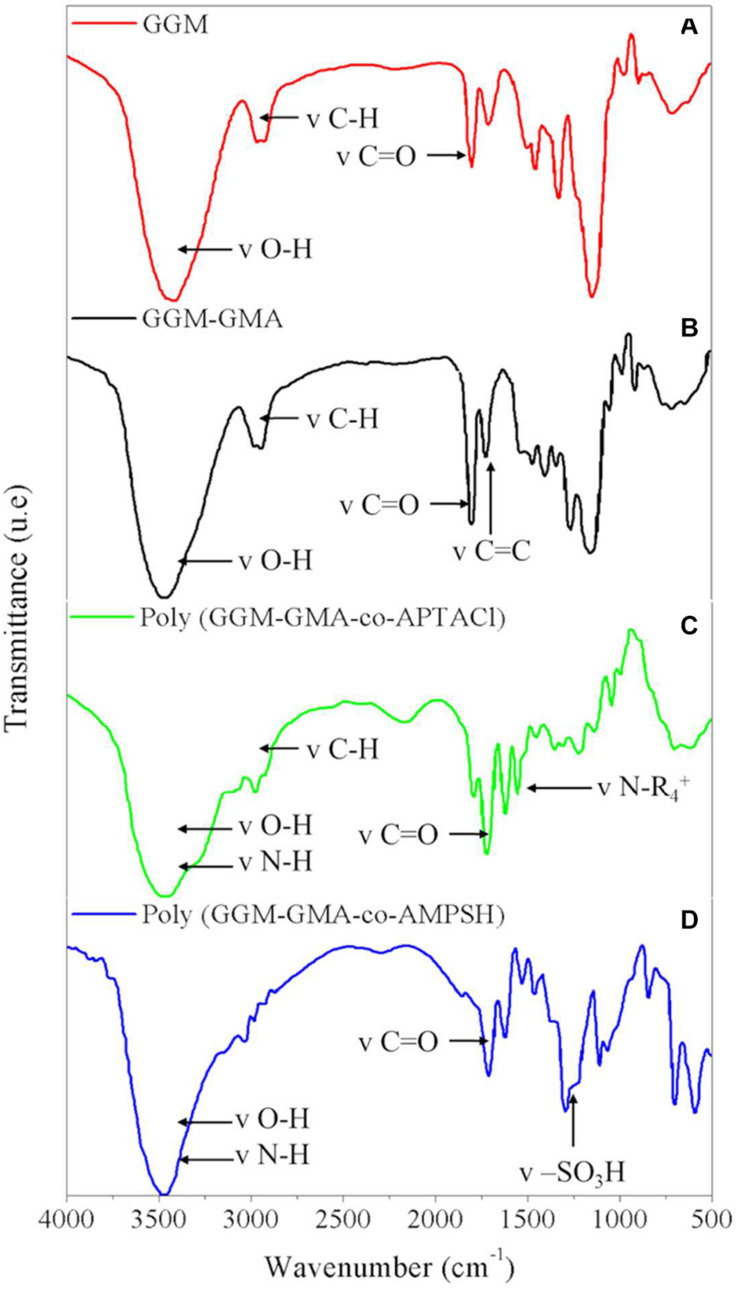
FT-IR of **(A)** GGM, **(B)** GGM-GMA, **(C)** poly(GGM-GMA/AMPTACl), and **(D)** poly(GGM-GMA/AMPSH). FT-IR, Fourier transform-infrared; GGM, *O*-acetyl galactoglucomannan; GMA, glycidyl methacrylate; poly(APTACl), poly[(3-acrylamidopropyl)trimethylammonium chloride].

Figure 2A shows the FT-IR spectra of GGM as the initial material, where the main characteristic bands are as follows: the signal at 3,408.80 cm^–1^ is attributed to the –OH stretching vibration, the strong signal at 1,734.39 cm^–1^ corresponds to the –C–O stretching vibration of the carboxyl group, and finally, the signal that appears at 2,929.70 cm^–1^ is attributed to CH_2_ groups. On the other hand, Figure 2B shows the representative signals of the GGM-GMA material, and it is possible to observe differences in the signals of the functional groups compared with those of GGM. The main signals of the functional group bands for GGM-GMA are the vibration signal corresponding to the C–C bond at 1,638.56 cm^–1^ (methacrylate group), which is more intense than that at the same value in GGM, and the signal at 1,378 cm^–1^, which indicates acetyl C–CH_3_. Other signals of interest for the characterization of the GGM-GMA material include 3,430.34 cm^–1^ (–OH), 2,890.78 cm^–1^ (C–H), 1,167.57 cm^–1^ (C–O–C asymmetric stretching signal), 1,055.65 cm^–1^ (C–O–C, hemicellulose and glycosidic bond signals), 883.97 cm^–1^ (anomeric carbons due to links with the β-glucoside at C-1), and 810.14 cm^–1^ (–CH bending signal of –CH groups). The signals at 1,520, 1,467, and 1,420 cm^–1^ associated with lignin are not observed in the FT-IR spectra of GGM-GMA. Signals between 909 and 945 cm^–1^ are not observed in the spectra; therefore, the presence of the GMA compound without chemical reaction is ruled out.

In the same way, the ion exchange hydrogels show differences in the FT-IR band signals of the functional groups compared with the spectrum obtained for the GGM-GMA precursor material. For analysis of the cationic hydrogel poly(GGM-GMA/APTACl), the FT-IR signals (see Figure 2C) display a peak for quaternary ammonium at 1,485.10 cm^–1^; 2,941.87 cm^–1^ represents the vibration of the CH_2_ methyl groups; 1,728.14 cm^–1^ corresponds to the ester functional group –C–O (both of the macromonomer and of the vinyl monomer); 3,440.35 cm^–1^ represents hydroxyl and N–H bond groups; and 1,059.28 cm^–1^ corresponds to –C–O–C of the glycosidic bond. On the other hand, when analyzing the anionic hydrogel poly(GGM-GMA/AMPSH), the FT-IR signals (see Figure 2D) display the following absorption bands: 3,196 cm^–1^ represents NH, 3,437 cm^–1^ represents (–OH), 1,645.58 cm^–1^ represents (–C–O), 1,036.43 cm^–1^ represents the glycosidic bond of GGM-GMA and to the (–SO) stretch, 1,224 cm^–1^ represents the (–SO_3_H group) attributed to the amide and sulfonic groups; and 2,930 cm^–1^ is the vibration of (Csp_3_–H). In addition, a band is observed at 619.55 cm^–1^, which indicates the stretching of the functional group (–SO), and for the secondary amide, there is a signal at 1,558.83 cm^–1^.

**FIGURE 3:**
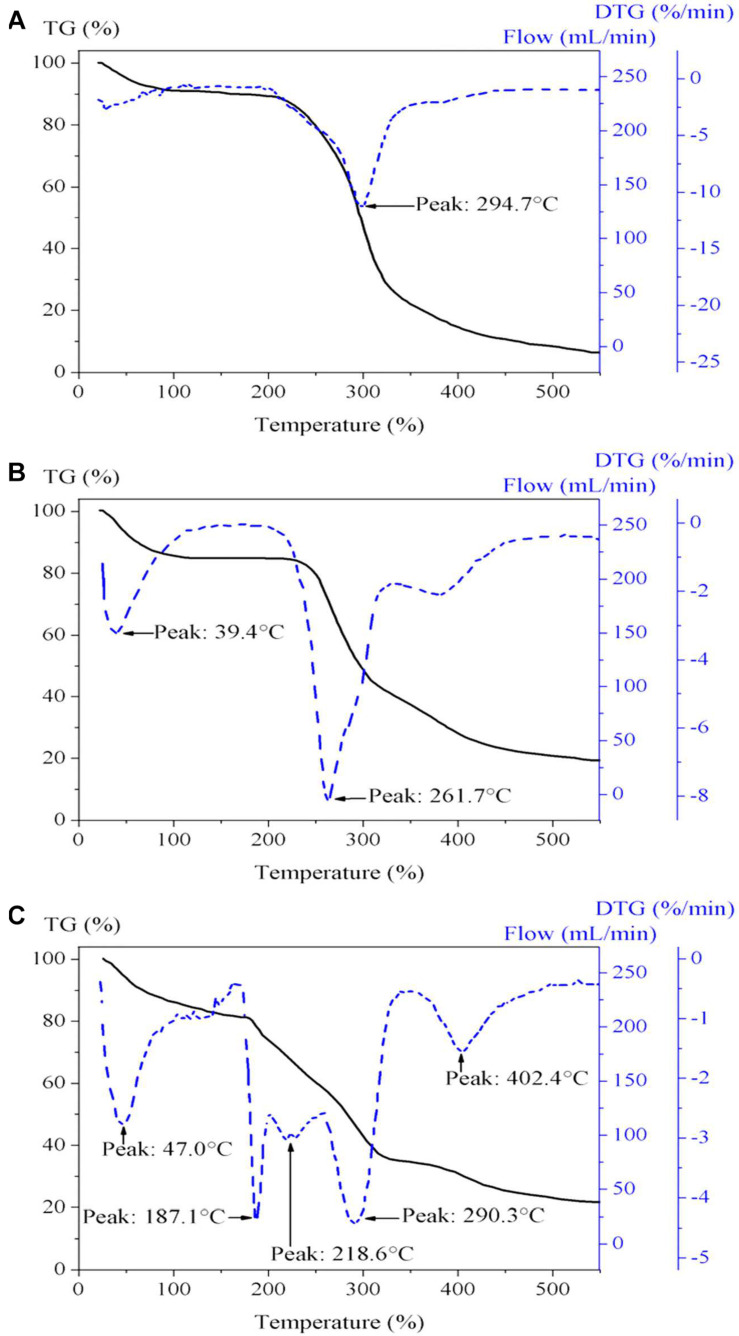
TGA-DTG of **(A)** GGM-MA, **(B)** poly(GGM-GMA/AMPTACl), and **(C)** poly(GGM-GMA/AMPSH). TGA, thermogravimetric analysis; DTG, derivative thermogravimetry; GGM, *O*-acetyl galactoglucomannan; GMA, glycidyl methacrylate; AMPSH, 2-acrylamido-2-methyl-1-propanesulfonic acid.

TGA–derivative thermogravimetry (DTG) analysis was performed for GGM-GMA and the synthesized ion exchange hydrogels. The thermogram of GGM-GMA is shown in Figure 3A, which indicates homogeneous decomposition of this material. In the temperature range between 40 and 220°C, a decrease of 10% in mass is observed, which is due to the release of the absorbed solvent and dehydration of the material. According to this result, it can be seen that the material is hygroscopic and that it absorbs moisture from the environment. GGM-GMA begins to decompose, significantly losing weight, at temperatures from approximately 210°C to approximately 330°C. According to experimental results reported in the literature (Al-Rudainy et al., 2019) on the properties of hemicellulose hydrogels, they indicate that 50% of the decomposition of the macromonomer is due to the decomposition of polysaccharides and branched GGM. From the thermogram of GGM-GMA, according to the DTG curve, 50% of the decomposition of the mass occurs at a temperature of 294.7°C. For GGM-GMA, at the end of the experiment, a residual mass percentage of 7.24% is obtained, which corresponds to the ash from the decomposition of all polysaccharides (Härdelin et al., 2020).

Figure 3B shows the results of the thermogram of the poly(GGM-GMA/APTACl) hydrogel, which presents a heterogeneous decomposition trend. The results show three temperature signals on the DTG curve, indicating the stages of the decomposition of the hydrogel. The first signal is at 39.4°C, which indicates the desorption of solvent and small molecules absorbed in the hydrogel structure and corresponds to 6.5% of the percentage of the loss of mass; the second signal occurs at 261.7°C, with 50% of the decomposition of the mass; and the third signal occurs at 380°C, with 35% of residual mass. The decomposition from 250 to 400°C was increased in the hydrogels containing poly(GGM-GMA/APTACl) compared with GGM due to losses of ammonia gas from the large-scale oxidative thermal decomposition of the network chains (Sahiner et al., 2015; Atta et al., 2017), which had a greater effect than decarboxylation of GGM.

Figure 3C shows the results of the thermogram for poly(GGM-GMA/AMPSH), which indicates the decomposition temperatures of its components. The curve presents a heterogeneous trend regarding the decomposition of the multiple components that make up the analysis mixture. According to the results of the DTG curve, the following signals can be observed at 47°C (Çavuş et al., 2016): evaporation of solvent molecules occurs, and a 12% loss in mass is recorded; in the temperature range from 100 to 187.1°C, dehydration of the material begins, which apparently absorbs water from the air. In the temperature range from 187.1 to 218.6°C, the decomposition of carbohydrate-based compounds and the breakdown of the crosslinking of the hydrogel occur. In the temperature range from 218.6 to 290.3°C, the complete decomposition of GGM-GMA and the decomposition of –SO_3_H groups (namely, SO_2_ and SO_3_) from the hydrogel occur; in the temperature range from 300 to 500°C, main chain degradation occurs, followed by crosslinking of bridges; and the decomposition temperature range at 180–400°C is attributed to ester bond breakdown in the structure of polymers.

**FIGURE 4:**
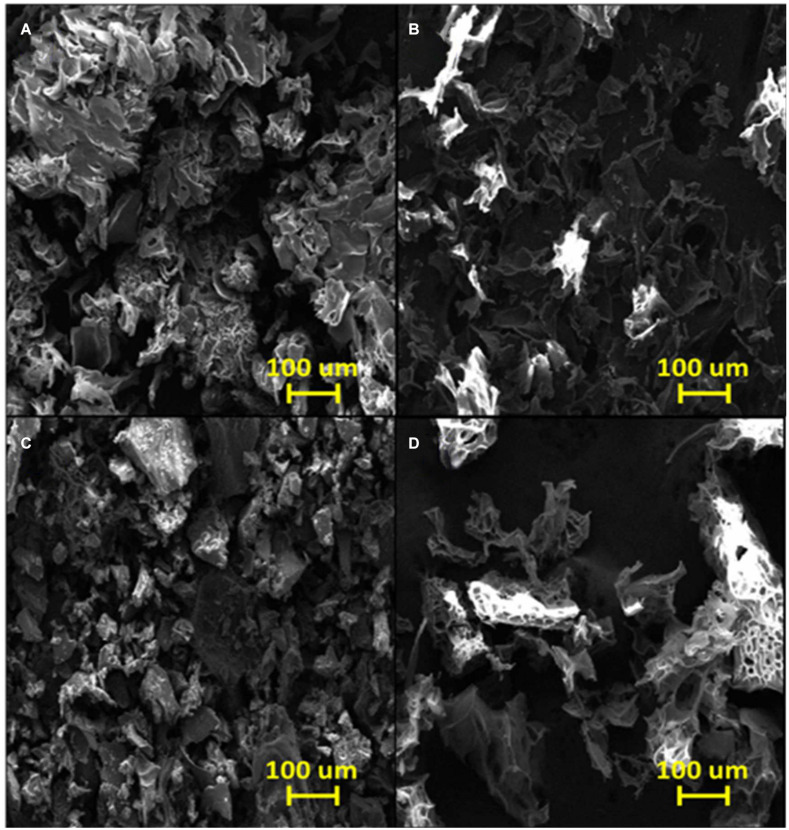
SEM images of **(A)** poly(GGM-GMA/APTACl), **(B)** poly(GGM-GMA/APTACl) + Cr(VI), **(C)** poly(GGM-GMA/AMPSH), and **(D)** poly(GGM-GMA/AMPSH) + Cu(II). SEM, scanning electron microscopy; GGM, *O*-acetyl galactoglucomannan; GMA, glycidyl methacrylate; APTACl, (3-acrylamidopropyl)trimethylammonium chloride; AMPSH, 2-acrylamido-2-methyl-1-propanesulfonic acid.

Figure 4A shows an SEM image of the poly(GGM-GMA/APTACl) hydrogel in which an amorphous structure is observed that possesses microcavities similar to those observed in sponges. In addition, the image shows that the material is massive with a large grain size, very similar to the structures of ion exchange resins with a high degree of crosslinking. According to the EDS results (see Table 1), it can be seen that the hydrogel has a high percentage of chlorine in the form of a chloride counterion present in the quaternary ammonium group. Figure 4B shows the microstructure of poly(GGM-GMA/APTACl) that was previously in a 100 mg L^–1^ chromate solution at basic pH. It can be seen that the material sample has amorphous particle sizes, and the structure tends to be in the form of thin tapes. On the other hand, the EDS results indicate that 10.94% weight is chromium absorbed (see Table 1). Figure 4C shows an irregular heterogeneous microstructure with small particle sizes. This structure corresponds to the poly(GGM-GMA/AMPSH) hydrogel. These hydrogel particles are highly porous, and the presence of sulfur, which is part of the sulfonate fixed functional group, can be seen in the EDS analysis results. In general, poly(GGM-GMA/AMPSH) has a different microstructure than poly(GGM-GMA/APTACl).

**TABLE 1:** EDS analysis of poly(GGM-GMA/APTACl) + Cr(VI), poly(GGM-GMA/APTACl) + As(V), and poly(GGM-GMA/AMPSH) + Cu(II).

	**Hydrogel**					
	**Poly(GGM-GMA/APTACl) + Cr(VI)**		**Poly(GGM-GMA/APTACl) + As(V)**		**Poly(GGM-GMA/AMPSH) + Cu(II)**	
**Element**	**Weight %**	**Atomic %**	**Weight %**	**Atomic %**	**Weight %**	**Atomic %**
C	47.94	58.35	51.80	64.34	19.92	48.22
N	7.68	8.01	12.38	13.19	0.77	1.59
O	33.44	30.56	20.74	19.34	5.55	10.09
Cl	–	–	0.55	0.23	–	–
As	–	–	14.52	0.23	–	–
Cr	10.94	3.08	–	–	–	–
S	–	–	–	–	14.13	12.81
Cu	–	–	–	–	59.63	27.29
Total	100.00		100.00		100.00	

*EDS, energy-dispersive spectroscopy; GGM, *O*-acetyl galactoglucomannan; GMA, glycidyl methacrylate; APTACl, (3-acrylamidopropyl) trimethylammonium chloride; AMPSH, 2-acrylamido-2-methyl-1-propanesulfonic acid.*

Figure 4D shows the morphology of poly(GGM-GMA/AMPSH) in a 100 mg L^–1^ copper(II) solution at acidic pH. The hydrogel sample in contact with copper generated a sponge-like porous structure. According to EDS analysis, 59.63% weight copper absorption was obtained (see Table 1).

It is probable that the synthesized hydrogels do not reach adequate mechanical and chemical stability, and they may dissolve and be completely destroyed in the presence of water. It is necessary to ensure that the crosslinking reaction between the GGM-GMA macromonomer and the anionic and cationic monomers is efficient and limits the excess of swelling.

### Water Uptake

The ion exchange hydrogels were swollen in deionized water for 24 h. A water absorption percentage of 33.9% was obtained for poly(GGM-GMA/AMPSH), while for poly(GGM-GMA/APTACl), a value of 28.5% was obtained. This small difference could be explained because poly(GGM-GMA/AMPSH) has polar groups such as carbonyl, hydroxyl, and esters and can weakly hydrolyze and acquire negative charges. These negatively charged groups can interact with the negative charge of the sulfonate group exerting intramolecular repulsive forces between the polymer chains of the crosslinked network. On the other hand, the positive charge of the quaternary ammonium groups has the capacity to neutralize to a certain extent to the negative charges of the polar groups carbonyl, hydroxyl, and esters of the GGM-GMA, causing the polymeric network not to swell excessively. Therefore, it is stated that poly(GGM-GMA/AMPSH) has a high degree of hydrophilicity. Elgueta et al. also obtained a similar result for a copolymer material of a GGM macromonomer crosslinked with an AMPSH monomer (Elgueta et al., 2016).

### Removal of Cu(II)

**FIGURE 5:**
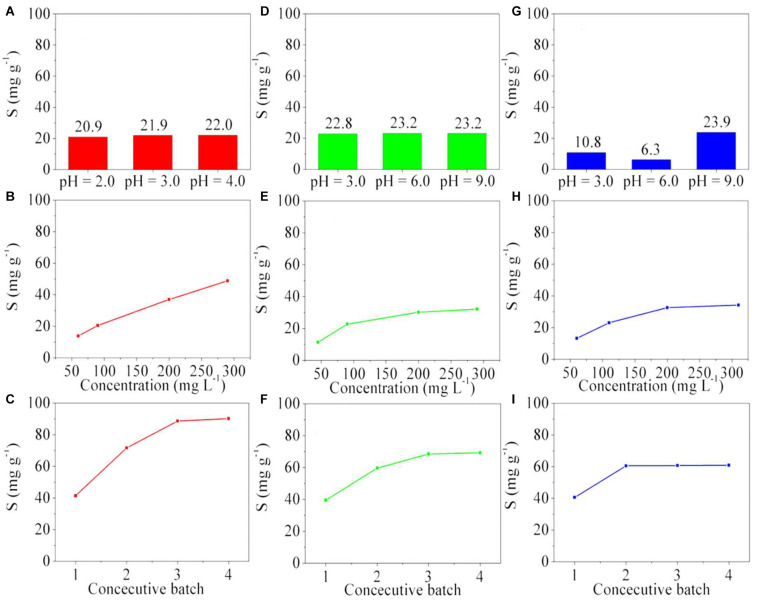
Absorption capacity of copper(II) **(A–C)** by poly(GGM-GMA/AMPSH) at **(A)** pH 2.0, 3.0, and 4.0 using 100 mg L^–1^ of copper(II). **(B)** different concentrations of copper(II) at pH 3.0. **(C)** consecutive batches using the same polymer sample at pH 3.0 and 200 mg L^–1^ of copper(II). Absorption capacity of Cr(VI) **(D–F)** by poly(GGM-GMA-*co*-APTACl) at **(D)** pH 3.0, 6.0, and 9.0 using 100 mg L^–1^ of Cr(VI). **(E)** different concentrations of Cr(VI) at pH 9.0. **(F)** Consecutive batches using the same polymer sample at pH 9.0 and 200 mg L^–1^ of Cr(VI). Absorption capacity of As(V) **(G–I)** by poly(GGM-GMA/APTACl) at **(G)** pH 3.0, 6.0, and 9.0 using 100 mg L^–1^ of As(V). **(H)** Different concentrations of As(V) at pH 9.0. **(I)** Consecutive batches using the same polymer sample at pH 9.0 and 200 mg L^–1^ of As(V). GGM, *O*-acetyl galactoglucomannan; GMA, glycidyl methacrylate; AMPSH, 2-acrylamido-2-methyl-1-propanesulfonic acid; APTACl, (3-acrylamidopropyl)trimethylammonium chloride.

Copper can generate divalent cations in aqueous solution, and depending on the concentration and pH value, it can change the ionic form or form coordination complexes with water or additional chelating agents. As copper in aqueous solution generates a stable divalent cation at pH values between 0.0 and 5.0, the poly(GGM-GMA/AMPSH) hydrogel is used because it has sulfonate anionic groups that interact with the positive charge of copper ions. Figure 5A shows the absorption capacity of copper ions by the poly(GGM-GMA/AMPSH) hydrogel when in a 100 mg L^–1^ copper(II) solution at pH values of 2.0, 3.0, and 4.0. The copper removal capacity is slightly influenced by pH. The maximum copper absorption was 22.9 mg g^–1^ at pH 4.0. However, the result at pH 3.0 was 21.9 mg g^–1^, which indicates that the influence of pH is almost null. Copper has the ability to generate various positively charged species [Cu^2+^, CuNO_3_^+^, CuOH^+^, Cu_2_(OH)_2_^2+^, and Cu_2_OH^3+^] capable of interacting more directly with the fixed groups of the hydrogel. No tests were carried out at pH values higher than 4.0 because divalent copper ions form complex species with different ionic charges or hydroxides that precipitate. At pH 2.0, the sulfonate functional group is moderately ionized, contracted, and packed for electrostatic interactions with copper cations. When the pH value increases, the sulfonate fixed functional groups become ionized, and the material swells because these groups repel due to their ionic charges. The resins that have sulfonate-fixed functional groups have better ion exchange performances at pH 3.0 because it has been shown that at this pH value, more than 85% of the sulfonate is completely ionized and available to perform electrostatic interactions and ion exchange reactions (Urbano and Rivas, 2013).

Figure 5B shows the absorption capacity of copper(II) by poly(GGM-GMA/AMPSH) at different copper concentrations in the feed at pH 3.0. The results indicate the progressive increase of the absorption capacity value when the concentration of the copper ion in the feed is increased. The high absorption capacity toward the divalent copper ion is due to electrostatic interactions between sulfonates and copper(II) cations. In this range of copper(II) concentrations, it was not possible to saturate the polymer. Figure 5C shows the results of consecutive batches using the same poly(GGM-GMA/AMPSH) sample at pH 3.0 and 200 mg L^–1^ of copper(II). After regenerative washing with brine, the divalent copper ion was desorbed from the polymer, and the polymer was ready for absorptive loading of the second cycle. The absorption capacity increased from 42 to 75 mg g^–1^. It is possible that the high concentration and ionic strength of the regenerating brine caused the hydrogel chains to stretch and relax their intramolecular forces; in this situation, the amount of fixed sulfonate groups could be increased for ion exchange. The absorption capacity increased and stabilized at a constant value between the third and fourth cycles, reaching values between 90 and 96 mg g^–1^, respectively.

### Removal of Cr(VI)

The removal of Cr(VI) oxyanions was evaluated with poly(GGM-GMA/APTACl). Figure 5D shows the results of the absorption capacity as a function of pH value using 100 mg L^–1^ of Cr(VI). It can be seen that 23.2 mg g^–1^ absorption was reached at pH values 6.0 and 9.0. In the case of pH 3.0, 22.8 mg g^–1^ absorption was achieved. These results can be attributed to the fact that the Cr(VI) species are present mostly as Cr_2_O_7_^2–^ and HCrO_4_^–^ oxyanions, depending on pH and concentration. The quaternary ammonium functional groups have high attraction for both bivalent and multivalent anions, and therefore, similar removal capacities can be observed in this pH range. Figure 5E shows the absorption capacity of Cr(VI) oxyanions by the poly(GGM-GMA/APTACl) hydrogel as a function of the chromium concentration in the feed at pH 9.0. It can be seen from the results that from 50 to 200 mg L^–1^ of Cr(VI), an increase occurs in the absorption capacity for this oxyanion. However, between 200 and 300 mg L^–1^, equilibrium is reached, and the absorption capacity of the poly(GGM-GMA/APTACl) hydrogel stabilizes at 33.3 mg g^–1^. Figure 5F shows the results of the maximum absorption capacity of Cr(VI) oxyanions by the poly(GGM-GMA/APTACl) hydrogel when successive cycles of loading and brine washing are performed. The loading was performed with 200 mg L^–1^ of Cr(VI) at pH 9.0. The results show an increase of the absorption capacity and the tendency to reach equilibrium in the fourth batch. The maximum absorption value reached is 69.2 mg g^–1^.

### Removal of As(V)

Figure 5G shows the maximum absorption capacity of As(V) by the poly(GGM-GMA/APTACl) hydrogel at different pH values using 100 mg L^–1^ of arsenic. It can be seen from the results that the absorption capacity of the arsenic oxyanions strongly depends on the pH of the aqueous medium, where the maximum capacity of 23.9 mg g^–1^ is achieved at pH 9.0. The results obtained occur because at pH 3.0, arsenate is present in aqueous medium as H_3_AsO_4_ and H_2_AsO_4_^–^; at pH 6.0, H_2_AsO_4_^–^ and HAsO_4_^2–^; and at pH 9.0, HAsO_4_^2–^ and AsO_4_^3–^. Figure 5H shows the results of the maximum absorption capacity at pH 9.0 of As(V) in the poly(GGM-GMA/APTACl) hydrogel, where the arsenic concentration in the feed is varied in the aqueous phase. These results indicate that an increase of the arsenic concentration produces an increase of the absorption capacity of the poly(GGM-GMA/APTACl) hydrogel. However, between 200 and 300 mg L^–1^, the absorption capacity reaches equilibrium, and the polymer is saturated. The quaternary ammonium functional groups of the hydrogel have a high affinity for divalent anions (HAsO_4_^2–^), but the monovalent anion (H_2_AsO_4_^–^) may compete to occupy these exchange sites and may displace divalent ions. This behavior can also be attributed to the fact that the chains of the hydrogel structure, which are mainly polysaccharides under slightly basic conditions, can be shrunk and packed, where the quaternary ammonium groups can also be balanced with OH^–^ ions. A similar result was obtained in the study of chromium oxyanions under the same pH value. Figure 5I shows the results of the absorption capacity of As(V) at pH 9.0 by the poly(GGM-GMA/APTACl) hydrogel when consecutive absorption cycles are carried out. The results show that the hydrogel material quickly reaches an equilibrium state of 60 mg g^–1^ after the second test cycle. Since there are two ionic species of arsenic, it is possible that at least one of the two species produces dipolar interactions with the nitrogen of the fixed quaternary ammonium groups and the oxygen of the oxyanion (Rivas et al., 2006).

### Removal of Metal Ions Using Polymer Blends and Multi-Ionic Solutions

**FIGURE 6:**
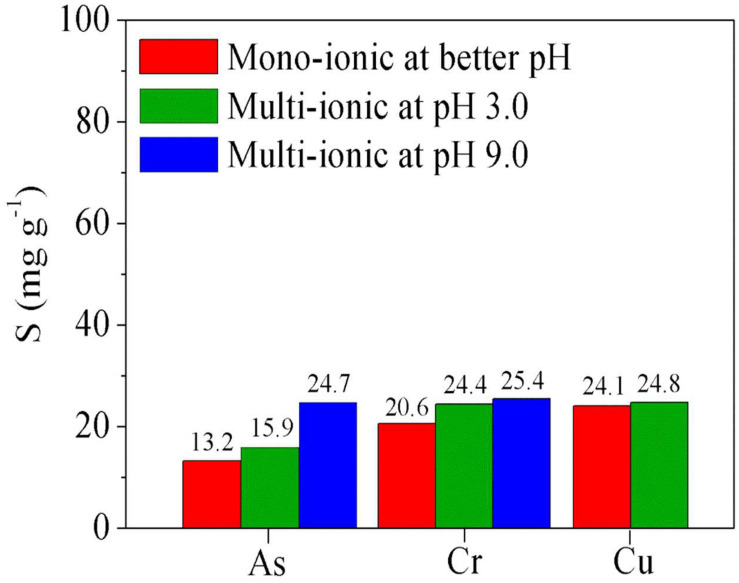
Absorption of metal ions using a polymer blend [poly(GGM-GMA/APTACl + poly(GGM-GMA/AMPSH)] from solutions: mono-ionic at better pH and concentration (red bars), multi-ionic solution at pH 3.0 (green bars), and multi-ionic solution at pH 9.0 (blue bars). GGM, *O*-acetyl galactoglucomannan; GMA, glycidyl methacrylate; APTACl, (3-acrylamidopropyl)trimethylammonium chloride; AMPSH, 2-acrylamido-2-methyl-1-propanesulfonic acid.

The experiments described in this section were carried out using polymer blends of poly(GGM-GMA/APTACl) + poly(GGM-GMA/AMPSH) to study their capacity to remove anions and cations from the same solution. Figure 6 (red bars) shows the results of the absorption of the metal ions from mono-ionic solutions using the polymer blend at the best removal pH. A high affinity for Cu(II) can be observed due to the multiple interactions that could result from the presence of the poly(GGM-GMA/AMPSH) hydrogel in the blend and its interactions with the ions in the solution. This behavior is similar to that obtained previously with individual hydrogels, indicating the higher removal of Cu(II).

The increase in the Cu(II) metal ion absorption capacity of the hydrogels can be explained by the metal ion binding ability of the formed hydrophilic (carbonyl, esters, hydroxyl, etc.) groups. In addition, copper can generate ionically charged aqueous complexes, which can interact through hydrogen bridges and dipolar interactions with the carbon chain structures of hydrogels. Similarly, Cr(VI) generates a good affinity for the polymer blend hydrogels, leading to efficient removal; however, low absorption of As(V) is achieved. It is possible that the interactions can be improved by altering the energy parameters and contact times between phases. Also the polyanion and polycation interactions do not dramatically decrease metal ion removal capacity. It is an advantage compared with previous results related to the use polycation and polyanion blends to remove metal and metalloid ions. The retention properties of arsenic ions from an aqueous solution by water-soluble cationic (polyquaternary ammonium) and anionic (polyacrylic acid) were investigated. Investigation showed the concerted action of polycations and polyanions on the ability to retain arsenic. The results showed a decrease in the ability of polycation when polyanion is present in the solution. This can be explained by an interaction of the COO^–^ group of the P(AA) with the N^+^(R_3_) groups of polycation by charge transfer that should have blocked both functional groups and the retention of arsenate is negligible (Rivas et al., 2006). In other research, poly(*N*-vinylpyrrolidone) (PVP) and poly(2-acrylamido-2-methylpropanesulfonate sodium) (PAMPS) were prepared and used for the removal of Cu^2+^, Cd^2+^, and Ni^2+^ ions. PAMPS exhibited a high retention capacity for all of the metal ions at both pH values studied. PVP exhibited selectivity for nickel ions. However, the homopolymer mixture containing PAMPS and PVP was inefficient for the retention of the studied metal ions. The electrostatic repulsion between macromolecules PAMPS probably promotes the formation of hydrogen bonds between the carbonyl groups of PVP and secondary amino groups of PAMPS. This favors the steric shielding of charged groups on the PAMPS, the occlusion of a high proportion of these groups in the PAMPS–PVP interpolymer complex, and the decrease of available carbonyl groups to coordinate metal ions (Valle et al., 2015).

In the current research, we found that polyanion and polycation interactions do not dramatically decrease metal ion removal capacity in mono-ionic solutions. It is probably due to the solid–liquid nature of the retention process. The polycation and polyanion interactions can be limited to the surface of the polymer hydrogels, which is different compared with polycation polyanion complex of water-soluble polymers.

Figure 6 (green bars) shows the results of the absorption of metal ions from a multi-ionic solution at pH 3.0 using a polymer blend. In these results, it can be seen that the absorption capacity is slightly improved, likely due to the fast interactions that occur among copper and sulfonic groups of poly(GGM-GMA/AMPSH) and simultaneously the interaction that occurs among chromium and arsenic with quaternary ammonium group of poly(GGM-GMA/APTACl, which stabilizes within the polymer mixture. Here, it is possible to note the similar behaviors of polyanion and polycation interactions, which do not dramatically decrease metal ion removal capacity in multi-ionic solutions.

On the other hand, in multi-ionic solutions, it is highly probable that the presence of the three ions with their respective speciations [Cu(II) (Cu^2+^, CuNO_3_^+^, CuOH^+^, and Cu_2_OH^3+^), Cr(VI) (HCrO_4_^–^, Cr_2_O_7_^2–^, and CrO_4_^2–^ and H_2_CrO_4_), and As(V) (H_3_AsO_4_, H_2_AsO_4_^–^, and HAsO_4_^2–^)] generates the same coordination and complexation mechanisms that occur when these ions are applied as wood preservatives. The chromate ions could be reduced by the action of the functional groups of the GGM and hydroxides and then Cr(III), aqueous complexes are formed. These Cr(III) ions interact with the As(V) and with the dissolved copper species [the formation of Cr(III)/As(V) cluster consisting of a Cr dimer bridged by an As(V) oxyanion, Cr(III), and Cu(II) complex with the wood components as well as hydroxide compounds] (Bull, 2001; Nico et al., 2004; Mohajerani et al., 2018).

Figure 6 (blue bars) shows the results of the absorption of Cr(VI) and As(V) from a multi-ionic solution at pH 9.0. It can be seen that the absorption capacity of As(V) increases. This behavior is due to the effect of pH, as well as the fact that the arsenic oxyanions could interact with chromium oxyanions through covalent bonds and interactions of hydrogen bridges, favoring the electrostatic interaction between the quaternary ammonium groups and the anions. Although the results obtained show a small difference, it is possible to note that there is competition for the quaternary ammonium functional groups between the chromate and arsenate ions. Moreover, these oxy-anions can form chelating bonds with the fixed groups composing the GGM structure.

## Conclusion

GGM hemicellulose was successfully modified through a transesterification reaction with GMA, generating a macromonomer (GGM-GMA) with the ability to be used as a macro-crosslinker in free-radical polymerization reactions. ^1^H-NMR spectroscopy reveals that it is possible to modify the GGM to obtain a GGM-GMA macromonomer. Results showed two peaks corresponding to the protons of the double bond –C=C– of the methacrylate group incorporated through the transesterification reaction.

Hydrogels with anion and cation exchange properties were synthesized successfully. The characterization demonstrated that the hydrogels possessed high hydrophilicity. FT-IR analysis showed that the GGM-GMA macromonomer was polymerized with the AMPSH and AMPTACl monomers, in which the signal corresponding to the alkene double bond disappears and the signals corresponding to the hydroxyl, quaternary ammonium, and sulfonate are intensified. TGA revealed the thermal stability of the hydrogels, and SEM/EDS was used to analyze the porous morphology and composition of the hydrogels.

The cationic hydrogel showed an efficient absorption capacity for Cr(VI) and As(V), and the anionic hydrogel reached the maximum absorption capacity for Cu(II). The absorption capacities were influenced by pH, concentration, and repeated use. In addition, the absorption of metal ions from multi-ionic solutions using polymer blends showed good results compared with the absorption from mono-ionic solutions. It is also important to observe that polyanion and polycation interactions do not dramatically decrease metal ion removal, and it is possible to design systems focused on the removal of cations and anions simultaneously.

## Data Availability Statement

The raw data supporting the conclusions of this article will be made available by the authors, without undue reservation.

## Author Contributions

JS: synthesis and characterization of hydrogels, metal removal, discussion of experiments, and manuscript writing. DD: contribution in FT-IR and NMR spectroscopy analysis, discussion, and manuscript writing. YT: analysis and discussion of TGA analysis. CX: analysis of SEM micrographs, discussion of GGM, and manuscript writing. SW: analysis and discussion of GGM and manuscript writing. All authors contributed to the article and approved the submitted version.

## Conflict of Interest

The authors declare that the research was conducted in the absence of any commercial or financial relationships that could be construed as a potential conflict of interest.
